# JNK3 Is Required for the Cytoprotective Effect of Exendin 4

**DOI:** 10.1155/2014/814854

**Published:** 2014-06-16

**Authors:** Hélène Ezanno, Valérie Pawlowski, Saida Abdelli, Raphael Boutry, Valery Gmyr, Julie Kerr-Conte, Christophe Bonny, François Pattou, Amar Abderrahmani

**Affiliations:** ^1^Lille 2 University, University of Lille Nord de France, European Genomic Institute for Diabetes, EGID FR 3508, UMR 8199, Lille, France; ^2^Department of Endocrine Surgery, Lille 2 University, University of Lille Nord de France, Lille University Hospital, INSERM UMR 859, Biotherapies for Diabetes, European Genomic Institute for Diabetes, Lille, France; ^3^Service of Medical Genetics, Centre Hospitalier Universitaire Vaudois (CHUV), University of Lausanne, 1011 Lausanne, Switzerland

## Abstract

Preservation of beta cell against apoptosis is one of the therapeutic benefits of the glucagon-like peptide-1 (GLP1) antidiabetic mimetics for preserving the functional beta cell mass exposed to diabetogenic condition including proinflammatory cytokines. The mitogen activated protein kinase 10 also called c-jun amino-terminal kinase 3 (JNK3) plays a protective role in insulin-secreting cells against death caused by cytokines. In this study, we investigated whether the JNK3 expression is associated with the protective effect elicited by the GLP1 mimetic exendin 4. We found an increase in the abundance of JNK3 in isolated human islets and INS-1E cells cultured with exendin 4. Induction of JNK3 by exendin 4 was associated with an increased survival of INS-1E cells. Silencing of JNK3 prevented the cytoprotective effect of exendin 4 against apoptosis elicited by culture condition and cytokines. These results emphasize the requirement of JNK3 in the antiapoptotic effects of exendin 4.

## 1. Introduction

Preservation of mechanisms underlying adaptation of beta cells mass and function is critical for glucose homeostasis, as the decline in functional beta cells mass is a key feature of the development of diabetes [1–5]. The incretin hormone glucagon-like peptide-1 (GLP1) plays an instrumental role in the control of beta cell mass and function [6–8]. Alteration of beta cell sensitivity to GLP1 is thought to contribute to the loss of functional beta cell mass in diabetes in both lean and obese individuals [9–11]. Beta cell abnormalities in the GLP1 sensitivity have been associated with a reduction in the GLP1 receptor expression in some animal models of diabetes [12, 13]. Administration of GLP1 improves beta cell survival in animal model of diabetes [14, 15]. A wealth of* in vitro* and* in vivo* studies show that this prosurvival effect is achieved by inhibiting beta cells apoptosis elicited by diabetogenic stressors such as proinflammatory cytokines [6, 16–23]. The effect achieved by the GLP1 and its analogs results from the activation of kinases and/or scaffold proteins, which in turn promote an antiapoptotic signaling cascade [6, 18, 19, 24–28]. One of the major kinases activated by GLP1 and its mimetic exendin 4 is the protein kinase B/AKT [16, 17, 27]. Activation of AKT by GLP1 results from the increased abundance of the insulin receptor substrate 2 (IRS2) [27]. In beta cells, the expression of IRS2 is controlled by the mitogen activated protein kinase 10 also called c-jun amino-terminal kinase 3 (JNK3) [29, 30]. Silencing of JNK3 by interference RNA dramatically reduces the IRS2 abundance in INS-1E cells [29]. As a result of JNK3 depletion an increase in cytokine-induced apoptosis ensues [29, 30]. In view of these data, the goal of this study was to investigate whether the JNK3 content is associated with beta cell protection achieved by the GLP1 mimetic exendin 4.

## 2. Material and Methods

### 2.1. Cell Culture, Human Islets Isolation, and Transfection

The rat insulin-secreting cell line INS-1E was maintained in RPMI 1640 medium supplemented with 10% fetal calf serum (FCS) (PAA laboratories, GE Healthcare, Velizy-Villacoublay, France), 1 mM sodium pyruvate, 50 *μ*M *β*-mercaptoethanol, and 10 mM Hepes [31]. Human pancreases were harvested from adult brain-dead donors in accordance with French regulations and with the local Institutional Ethical Committee from the “Centre Hospitalier Régional et Universitaire de Lille.” Pancreatic islets were isolated after ductal distension of the pancreas and digestion of the tissue as described previously [32]. All experiments were carried out at least on islets with a purity of and viability >80%. Purified islets were cultured in CMRL 1066 medium (Gibco BRL, Life Technologies) containing 0.625% free fatty acid human serum albumin (Roche Diagnostics), penicillin (100 *μ*UI/mL), and streptomycin (100 *μ*g/mL). The siRNA duplexes directed against JNK3 (siJNK3) or siRNA control against GFP (siGFP) were previously described [29–31]. The siRNA duplexes were introduced using the Lipofectamine 2000 (Life Technology, Saint Aubin, France) as described [29, 30].

### 2.2. Western Blotting Experiments

INS-1E and isolated human islets cells were scrapped in cold PBS buffer and cell pellets were incubated 30 min on ice in lysis buffer (20 mM Tris acetate pH 7, 0.27 mM sucrose, 1% Triton X-100, 1 mM EDTA, and 1 mM EGTA, 1 mM DTT) supplemented with antiproteases and antiphosphatases (Roche, Meylan, France). Cell lysates were centrifugated 15 min at 18,000 g and supernatants were used to analyze proteins. Protein extracts were solubilized in Laemmli buffer (40% glycerol, 20% *β*-mercaptoethanol, 8% SDS, 0.02% bromophenol blue, 0.25 mM Tris-HCl, pH 6.8) and denatured 10 min at 95°C before loading onto the gel. Proteins were separated on 10% SDS-polyacrylamide gel and electrically blotted to nitrocellulose membranes. The proteins were detected after an overnight incubation of the membrane at 4°C with the specific primary antibodies against JNK3 (dilution 1 : 1000; Cell Signaling Technology, MA, USA), JNK2 (dilution 1 : 1000; Cell Signaling Technology, MA, USA), *β*-actin (1 : 5000; Sigma, Saint Quentin, France), or *α*-tubulin (1 : 5000; Sigma, Saint Quentin, France), diluted in buffer containing 0.1% Tween 20 with either 2% milk (for JNK3) or 5% BSA (for JNK2) or 5% milk (for *β*-actin and *α*-tubulin). Proteins were visualized with IRDye800 or IRDye700 (Eurobio, Les Ulis, France) as secondary antibodies. Quantification was performed using the Odyssey infrared imaging system (Eurobio) [29, 30].

### 2.3. Apoptosis Assay

Apoptosis was evaluated in cells transfected with the siRNAs and exposed to a cytokine cocktail (R&D Systems, Minneapolis, MN, USA) of rat IL-1*β* (10 ng/mL), mouse TNF*α* (25 ng/mL), and rat IFN*γ* (150 ng/mL) for 24 h. Apoptosis was determined by scoring cells displaying pycnotic nuclei (visualized with Hoechst 33342) [31]. The counting was performed blindly by three different experimenters.

### 2.4. Statistical Analysis

ANOVA was used for statistical significance, followed by the post hoc Bonferroni test (Dunnett's test) when experiments included more than two groups. The level of significance was set at *P* < 0.05 (SAS statistical package; SAS, Carry, NC).

## 3. Results 

### 3.1. Exendin 4 Increases the JNK3 Abundance in Isolated Human Islets and INS-1E

**Figure 1:**
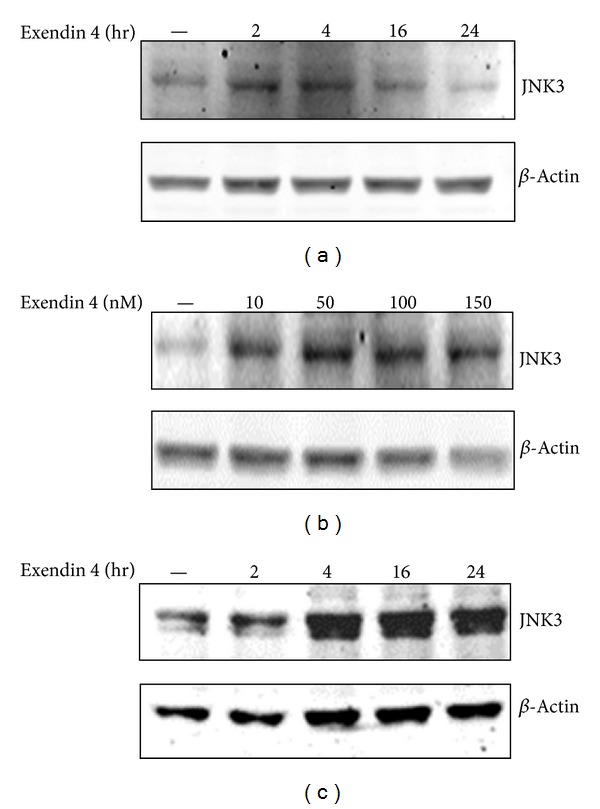
The effect of exendin 4 on the JNK3 content. JNK3 abundance in (a) isolated human islets (from three different donors) cultured with 50 nM exendin 4 for the indicated times or (b) with different exendin 4 concentrations for 4 hrs. (c) INS-1E cells cultured with 50 nM exendin 4 for the indicated times. For western blotting experiments, protein extracts (50 *μ*g) were loaded into a polyacrylamide gel electrophoresis. Immunoblotting was achieved using the anti-JNK3 and anti-*β*-actin antibodies. The data is one representative experiment out of three.

Several studies, including ours, have shown that the GLP1 receptor agonists prevent apoptosis elicited by prolonged exposure with cytokines [21, 23, 31]. Typically, the cytoprotective effect of the GLP1 mimetic is achieved through induction of key prosurvival proteins [6, 25]. In this regard we questioned whether exendin 4 increased the abundance of JNK3. We found that exposure of isolated human islets cells to exendin 4 elevated the JNK3 abundance as revealed by western blotting analysis (Figure 1(a)). The increase in the JNK3 protein started as early as 2 hr and declined after 4 hr (Figure 1(a)). Induction of JNK3 by exendin 4 was observable at 10 nM but was optimal at 50 nM (Figure 1(b)). Western blotting experiment confirmed elevation of JNK3 protein by exendin 4 in INS-1E cells (Figure 1(c)). However, induction of JNK3 by the GLP1 receptor agonist came later (after 4 hr of incubation) and persisted until 24 h treatment (Figure 1(c)).

### 3.2. JNK3 Is Required for the Cytoprotective Effects of Exendin 4 in INS-1E Cells

**Figure 2:**
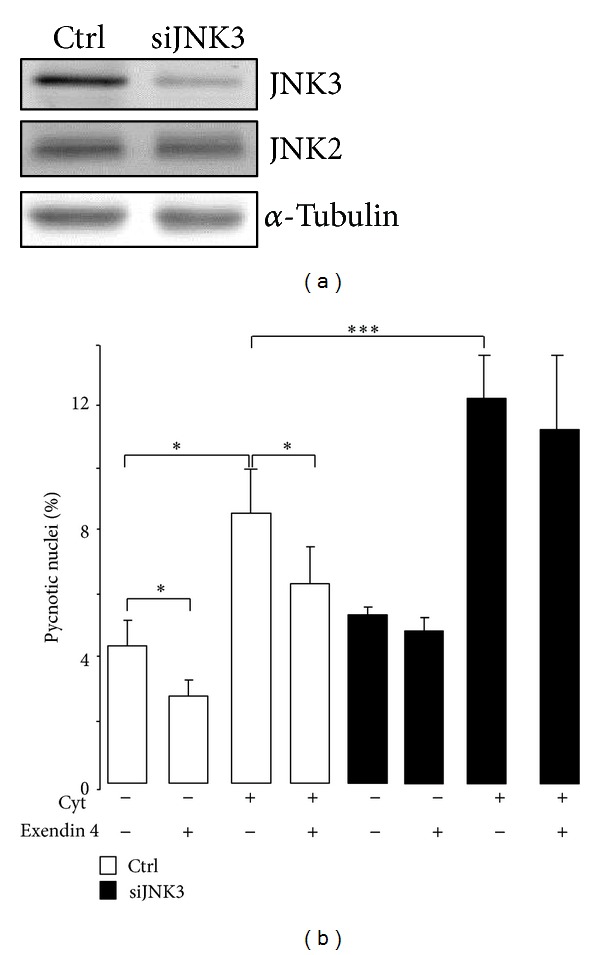
Impact of the JNK3 silencing on the protective effects of exendin 4. INS-1E cells were transfected with the siRNA duplex directed against JNK3 (siJNK3) or control siRNA (siGFP, Ctrl). (a) For western blotting analysis of the JNK3 level, total proteins were prepared 48 hr after transfection. Immunoblotting was done using the anti-JNK3, anti-JNK2, and anti-*α*-tubulin antibodies (b) for scoring death; the cells were preincubated 24 hr after transfection with 50 nM exendin 4 for 8 hr. The rate of apoptosis was scored by counting pycnotic nuclei in INS-1E cells exposed for 16 hr to the cocktail of cytokines including 10 ng/mL IL-1*β*, 15 ng/mL TNF*α*, and 150 ng/mL IFN*γ*. Results are expressed as mean ± SEM of 3 independent experiments. *P < 0.05; ***P < 0.001.

We next investigated whether JNK3 was required for the cytoprotective effect of exendin 4. To this end, INS-1E cells were transfected with the duplex siRNAs directed against JNK3 mRNA (siJNK3) [29, 30]. The latter efficiently and selectively silenced the expression of JNK3 in INS-1E cells (Figure 2(a)). As anticipated, incubation of the cells with cytokines for 24 hr elicited a 2-fold increase in apoptosis (Figure 2(b)). Exendin 4 efficiently reduced death evoked by culture conditions and cytokines (Figure 2(b)). As previously observed [29, 30], silencing of JNK3 potentiated cytokines-induced apoptosis (Figure 2(b)). In addition, diminution of JNK3 by siJNK3 abolished the protective effects accomplished by exendin 4 (Figure 2(b)). These data point out that JNK3 levels are pivotal for the coupling of exendin 4 and protection of cells against apoptosis evoked by cytokines.

## 4. Discussion

There is a growing body of evidence that the GLP1 and its mimetics trigger cytoprotective effects on beta cells by stimulating the abundance of antiapoptotic proteins [6, 17, 25–27, 31, 33]. Several reports have now delineated a role for JNK3 as a key player in protecting beta cells against apoptosis [29, 30]. A hallmark of this claim is that selective silencing of JNK3 increases apoptosis induced by cytokines [29, 30]. Inversely, we questioned whether the JNK3 content could be stimulated by the GLP1 mimetic exendin 4. We found that exposure of isolated human islets to exendin 4 increases the JNK3 protein content. Although the antiapoptotic mechanisms activated by the GLP1 mimetics are globally similar between human islets and the rat insulin-secreting INS-1E cells [6, 17, 25, 26, 31], the spatial and temporal regulation of certain pathways evoked by GLP1 and its analogs may be species-specific. Different temporal activation of the antiapoptotic ERK pathway between isolated human islets and INS-1E cells has been shown in response to RF26a RFamide peptide [34]. While ERK is activated by the peptide in human islets and INS-1E cells, peptide-induced ERK activation is more prolonged in INS-1E cells [35]. One study has shown that beta cells behaviour in response to GLP1 is different between human and rodent islets [36]. GLP1 promotes cooperation and connectedness between beta cells within human islets whereas it does not do this in rodent cells [36]. This difference may elicit some changes in the spatial and temporal regulation of genes expression and pathways. In this regard, we observed that induction of JNK3 content by exendin 4 was faster and declined more rapidly in human islets when compared to INS-1E cells.

The induction of JNK3 in human islets and INS-1E cells led us to ask whether such phenomenon contributed to the protective effects elicited by exendin 4 against apoptosis. One clue was that the increase of JNK3 content in cultures of INS-1E cells for 24 h was associated with a significant reduction in apoptosis under normal culture condition. This antiapoptotic effect achieved by the GLP1 mimetic was abolished when JNK3 content was reduced by siRNA. We have previously published that cytokine treatment of INS-1E cells with cytokines worsens death caused by apoptosis [31]. The rise of death induced by cytokines is alleviated by coculturing the cells with exendin 4 [31]. The experiments unveiled that protection of INS-1E cells by exendin 4 against cytokine-induced apoptosis is abolished when the JNK3 abundance is attenuated.

Several key transcription factors and signalling proteins including protein kinase A (PKA), PKB/AKT, PKC-zeta, ERK, endoplasmic reticulum stress, and epidermal growth factor receptor are involved in the cytoprotective effects achieved by the GLP1 mimetics [6, 18, 19, 24–26]. Abdelli and coauthors have shown a reduction in the expression of insulin receptor substrate 2 (IRS2) and Akt activation upon silencing of JNK3 [29, 30]. However, JNK3 is mainly localized in the nucleus of beta cells [29, 30], suggesting that IRS2 cannot be the only target of the kinase. Future studies are needed to identify other targets of JNK3 that are required for the antiapoptotic effects of exendin 4. Such investigation could uncover novel protective pathways of beta cells and eventually lead to innovative antidiabetic therapeutic targets.

## References

[1] Prentki M, Nolan CJ (2006). Islet *β* cell failure in type 2 diabetes. *Journal of Clinical Investigation*.

[2] Weir GC, Bonner-Weir S (2004). Five of stages of evolving *β*-cell dysfunction during progression to diabetes. *Diabetes*.

[3] Rahier J, Guiot Y, Goebbels RM, Sempoux C, Henquin JC (2008). Pancreatic *β*-cell mass in European subjects with type 2 diabetes. *Diabetes, Obesity and Metabolism*.

[4] Camastra S, Manco M, Mari A (2005). *β*-cell function in morbidly obese subjects during free living: long-term effects of weight loss. *Diabetes*.

[5] Ravier MA, Leduc M, Richard J (2014). *β*-Arrestin2 plays a key role in the modulation of the pancreatic beta cell mass in mice. *Diabetologia*.

[6] Drucker DJ (2006). The biology of incretin hormones. *Cell Metabolism*.

[7] Ahlkvist L, Brown K, Ahren B (2013). Upregulated insulin secretion in insulin-resistant mice: evidence of increased islet GLP1 receptor levels and GPR119-activated GLP1 secretion. *Endocrine Connections*.

[8] Bennett BL, Sasaki DT, Murray BW (2001). SP600125, an anthrapyrazolone inhibitor of Jun N-terminal kinase. *Proceedings of the National Academy of Sciences of the United States of America*.

[9] Kjems LL, Holst JJ, Vølund A, Madsbad S (2003). The influence of GLP-1 on glucose-stimulated insulin secretion: effects on *β*-cell sensitivity in type 2 and nondiabetic subjects. *Diabetes*.

[10] Schäfer SA, Tschritter O, Machicao F (2007). Impaired glucagon-like peptide-1-induced insulin secretion in carriers of transcription factor 7-like 2 (TCF7L2) gene polymorphisms. *Diabetologia*.

[11] Holst JJ, Knop FK, Vilsbøll T, Krarup T, Madsbad S (2011). Loss of incretin effect is a specific, important, and early characteristic of type 2 diabetes. *Diabetes Care*.

[12] Xu G, Kaneto H, Laybutt DR (2007). Downregulation of GLP-1 and GIP receptor expression by hyperglycemia: possible contribution to impaired incretin effects in diabetes. *Diabetes*.

[13] Shu L, Matveyenko AV, Kerr-Conte J, Cho J-H, McIntosh CHS, Maedler K (2009). Decreased TCF7L2 protein levels in type 2 diabetes mellitus correlate with downregulation of GIP- and GLP-1 receptors and impaired beta-cell function. *Human Molecular Genetics*.

[14] Farilla L, Hongxiang H, Bertolotto C (2002). Glucagon-like peptide-1 promotes islet cell growth and inhibits apoptosis in Zucker diabetic rats. *Endocrinology*.

[15] Wang Q, Brubaker P (2002). Glucagon-like peptide-1 treatment delays the onset of diabetes in 8 week-old db/db mice. *Diabetologia*.

[16] Jhala US, Canettieri G, Screaton RA (2003). cAMP promotes pancreatic *β*-cell survival via CREB-mediated induction of IRS2. *Genes and Development*.

[17] Cornu M, Yang J-Y, Jaccard E, Poussin C, Widmann C, Thorens B (2009). Glucagon-like peptide-1 protects *β*-cells against apoptosis by increasing the activity of an Igf-2/Igf-1 receptor autocrine loop. *Diabetes*.

[18] Buteau J, Roduit R, Susini S, Prentki M (1999). Glucagon-like peptide-1 promotes DNA synthesis, activates phosphatidylinositol 3-kinase and increases transcription factor pancreatic and duodenal homeobox gene 1 (PDX-1) DNA binding activity in beta (INS-1)- cells. *Diabetologia*.

[19] Brubaker PL, Drucker DJ (2004). Minireview: Glucagon-like peptides regulate cell proliferation and apoptosis in the pancreas, gut, and central nervous system. *Endocrinology*.

[20] Xu G, Stoffers DA, Habener JF, Bonner-Weir S (1999). Exendin-4 stimulates both *β*-cell replication and neogenesis, resulting in increased *β*-cell mass and improved glucose tolerance in diabetic rats. *Diabetes*.

[21] Natalicchio A, de Stefano F, Orlando MR (2010). Exendin-4 prevents c-Jun N-terminal protein kinase activation by Tumor Necrosis Factor-*α* (TNF*α*) and inhibits TNF*α*-induced apoptosis in insulin-secreting cells. *Endocrinology*.

[22] Favre D, Niederhauser G, Fahmi D (2011). Role for inducible cAMP early repressor in promoting pancreatic beta cell dysfunction evoked by oxidative stress in human and rat islets. *Diabetologia*.

[23] Li L, El-Kholy W, Rhodes CJ, Brubaker PL (2005). Glucagon-like peptide-1 protects beta cells from cytokine-induced apoptosis and necrosis: role of protein kinase B. *Diabetologia*.

[24] Buteau J, Spatz ML, Accili D (2006). Transcription factor FoxO1 mediates glucagon-like peptide-1 effects on pancreatic *β*-cell mass. *Diabetes*.

[25] Yusta B, Baggio LL, Estall JL (2006). GLP-1 receptor activation improves *β* cell function and survival following induction of endoplasmic reticulum stress. *Cell Metabolism*.

[26] Dalle S, Quoyer J, Varin E, Costes S (2011). Roles and regulation of the transcription factor CREB in pancreatic *β*-cells. *Current Molecular Pharmacology*.

[27] Park S, Dong X, Fisher TL (2006). Exendin-4 uses Irs2 signaling to mediate pancreatic *β* cell growth and function. *Journal of Biological Chemistry*.

[28] Abderrahmani A, Cheviet S, Ferdaoussi M, Coppola T, Waeber G, Regazzi R (2006). ICER induced by hyperglycemia represses the expression of genes essential for insulin exocytosis. *EMBO Journal*.

[29] Abdelli S, Bonny C (2012). JNK3 maintains expression of the insulin receptor substrate 2 (IRS2) in insulin-secreting cells: functional consequences for insulin signaling. *PLoS ONE*.

[30] Abdelli S, Puyal J, Bielmann C (2009). JNK3 is abundant in insulin-secreting cells and protects against cytokine-induced apoptosis. *Diabetologia*.

[31] Ferdaoussi M, Abdelli S, Yang J-Y (2008). Exendin-4 protects *β*-cells from interleukin-1*β*-induced apoptosis by interfering with the c-Jun NH2-terminal kinase pathway. *Diabetes*.

[32] Vantyghem M-C, Kerr-Conte J, Arnalsteen L (2009). Primary graft function, metabolic control, and graft survival after islet transplantation. *Diabetes Care*.

[33] Wang L-X, Wang Y-P, Chen Z (2010). Exendin-4 protects murine pancreatic *β*-cells from dexamethasone-induced apoptosis through PKA and PI-3K signaling. *Diabetes Research and Clinical Practice*.

[34] Chandrasekera PC, Pippin JJ (2014). Of rodents and men: species-specific glucose regulation and type 2 diabetes research. *Altex*.

[35] Granata R, Settanni F, Trovato L (2014). RFamide peptides 43RFa and 26RFa both promote survival of pancreatic beta-cells and human pancreatic islets but exert opposite effects on insulin secretion. *Diabetes*.

[36] Hodson DJ, Mitchell RK, Bellomo EA (2013). Lipotoxicity disrupts incretin-regulated human beta cell connectivity. *The Journal of Clinical Investigation*.

